# A Novel Ferroptosis-Based Molecular Signature Associated with Biochemical Recurrence-Free Survival and Tumor Immune Microenvironment of Prostate Cancer

**DOI:** 10.3389/fcell.2021.774625

**Published:** 2022-01-06

**Authors:** Zhi-Bin Ke, Qi You, Jiang-Bo Sun, Jun-Ming Zhu, Xiao-Dong Li, Dong-Ning Chen, Li Su, Qing-Shui Zheng, Yong Wei, Xue-Yi Xue, Ning Xu

**Affiliations:** ^1^ Department of Urology, Urology Research Institute, The First Affiliated Hospital, Fujian Medical University, Fuzhou, China; ^2^ Department of Radiotherapy, The First Affiliated Hospital of Fujian Medical University, Fuzhou, China; ^3^ Fujian Key Laboratory of Precision Medicine for Cancer, The First Affiliated Hospital, Fujian Medical University, Fuzhou, China

**Keywords:** prostate cancer, ferroptosis, biochemical recurrence, tumor immune microenvironment, molecular signature

## Abstract

**Objective:** To identify ferroptosis-related molecular clusters, and to develop and validate a ferroptosis-based molecular signature for predicting biochemical recurrence-free survival (BCRFS) and tumor immune microenvironment of prostate cancer (PCa).

**Materials and Methods:** The clinical data and transcriptome data of PCa were downloaded from TCGA and GEO database. Ferroptosis-related genes (FRGs) were obtained from FerrDb database. We performed consensus clustering analysis to identify ferroptosis-related molecular subtypes for PCa. Univariate and multivariate Cox regression analysis were used to establish a ferroptosis-based signature for predicting BCRFS. Internal verification, external verification and subgroup survival analysis were then successfully performed.

**Results:** There was a total of 40 differentially expressed FRGs in PCa. We then identified three ferroptosis-related molecular clusters of PCa, which have significantly different immune infiltrating cells, tumor immune microenvironment and PD-L1 expression level. More importantly, a novel ferroptosis-based signature for predicting BCRFS of PCa based on four FRGs (including ASNS, GPT2, NFE2L2, RRM2) was developed. Internal and external verifications were then successfully performed. Patients with high-risk score were associated with significant poor BCRFS compared with those with low-risk score in training cohort, testing cohort and validating cohort, respectively. The area under time-dependent Receiver Operating Characteristic (ROC) curve were 0.755, 0.705 and 0.726 in training cohort, testing cohort and validating cohort, respectively, indicating the great performance of this signature. Independent prognostic analysis indicated that this signature was an independent predictor for BCRFS of PCa. Subgroup analysis revealed that this signature was particularly suitable for younger or stage T III-IV or stage N0 or cluster 1-2 PCa patients. Patients with high-risk score have significantly different tumor immune microenvironment in comparison with those with low-risk score. The results of qRT-PCR successfully verified the mRNA expression levels of ASNS, GPT2, RRM2 and NFE2L2 in DU-145 and RWPE-1 cells while the results of IHC staining exactly verified the relative protein expression levels of ASNS, GPT2, RRM2 and NFE2L2 between PCa and BPH tissues.

**Conclusions:** This study successfully identified three ferroptosis-related molecular clusters. Besides, we developed and validated a novel ferroptosis-based molecular signature, which performed well in predicting BCRFS and tumor immune microenvironment of PCa.

## Introduction

As the most prevalent non-cutaneous carcinoma of males in the world, prostate cancer (PCa) is considered as the fifth leading cause of death (Xu et al., 2020a; Huang et al., 2020). Radical prostatectomy (RP) remains the standard curable strategy for localized PCa; however, there is still a certain proportion of patients developing biochemical recurrence (BCR) (Venclovas et al., 2019), which indicated a possibility of underlying clinical metastases and poor prognosis (Xu et al., 2016; Xu et al., 2020b). The early recognition of BCR is of great significance for the subsequent treatment strategies of PCa patients. Although several studies have investigated diverse biomarkers or clinical factors to predict BCR, there are no recognized molecular subtypes and prognostic signature related to BCR for PCa (Arora and Barbieri, 2018).

Recently, immunotherapy has been regarded as an indispensable treatment modality for malignancies (Riley et al., 2019). With the Food and Drug Administration (FDA) approvals of sipuleucel-T into PCa treatment, immunotherapy has improved outcomes of carefully selected patients (Bilusic et al., 2017; Milonas et al., 2019). Furthermore, several studies have revealed that immune responses and clinical responses could be tremendously promoted by combining various immunotherapeutic agents with hormonal therapy, chemotherapy or DNA-damaging agents (Bilusic et al., 2017; Gamat and McNeel, 2017). Immunotherapy showed a promising future in patients with PCa (Bilusic et al., 2017). Therefore, in addition to discovering the optimal treatment combination, there is an urgent need to develop potential biomarkers for immunotherapy response and tumor immune microenvironment.

Ferroptosis is considered a programmed cell death pattern, which is characterized by iron-dependent peroxidation (Zuo et al., 2020). Increased cellular activity of carcinoma cells leads to high susceptibility to ferroptosis (Kwon et al., 2020). In recent years, studies related to the role of ferroptosis in carcinogenesis and progression have gained momentum (Stockwell et al., 2017). PCa has been recognized as one of the ferroptosis-related diseases (Ghoochani et al., 2021). Previous studies have demonstrated that the abnormal lipid homeostasis is associated with tumorigenesis of PCa and progression to castration-resistant prostate cancer (Blomme et al., 2020). However, to our knowledge, studies focusing on the correlation of ferroptosis with biochemical recurrence and anti-tumor immunology of PCa were rather limited.

In this study, we identified three ferroptosis-related molecular clusters for PCa. Moreover, interestingly, we successfully developed a ferroptosis-based signature for predicting biochemical recurrence-free survival (BCRFS) of PCa. Internal and external verifications were then resoundingly performed. Besides, the correlations of the ferroptosis-related molecular clusters and signature with tumor immune microenvironment, cancer stemness and drug sensitivity were particularly explored. We then conducted qRT-PCR in DU-145 and RWPE-1 cells and immunohistochemical (IHC) staining in PCa and BPH tissues to validate the mRNA and protein expression levels of four risk FRGs.

## Materials and Methods

### Data Collection

This study was approved by the Institutional Ethics Committee of First Affiliated Hospital of Fujian Medical University and was conducted in accordance with 1964 Helsinki declaration and its later amendments. Written informed consent was provided by all included patients before sample collection. Fresh postoperative PCa tissues and benign prostatic hyperplasia (BPH) tissues were obtained from three PCa patients and three BPH patients who have been pathologically diagnosed by biopsy and were treated in the Department of Urology, the First Affiliated Hospital of Fujian Medical University.

Transcriptome profiles of 499 PCa cases and 52 normal cases were downloaded from the Cancer Genome Atlas (TCGA) database (https://portal.gdc.cancer.gov/). In this study, considering that there were only 70 samples that have the data type of “days_to_first_biochemical_recurrence”, we combined “biochemical_recurrence” and “A8_New_Event_Type” as the BCR status, and combined “days_to_first_biochemical_recurrence” and “A8_New_Event_Time” as the time to BCR. (1) In the 70 samples that have data type of “days_to_first_biochemical_recurrence”, we used “days_to_first_biochemical_recurrence” as the time to BCR and used “biochemical_recurrence” as the BCR status. (2) In those that did not have data type of “days_to_first_biochemical_recurrence”, we utilized “A8_New_Event_Time” as the time to BCR. (3) If we utilized “A8_New_Event_Time” as the time to BCR, we preferentially used “A8_New_Event_Type” as the BCR status; in those without data of “A8_New_Event_Type”, we used “biochemical_recurrence” as the BCR status. BCR-free survival (BCRFS) was defined as the interval from radical treatment to first BCR or death.

A total of 405 PCa cases in TCGA database had complete data of transcriptome, BCR status and BCR-free time. Dataset of GSE70770 (including 203 PCa cases with complete data of transcriptome, BCR status and BCR-free time) was obtained from Gene Expression Omnibus (GEO) database (https://www.ncbi.nlm.nih.gov/geo/).

FerrDb database (http://www.zhounan.org/ferrdb/) is the world’s first database related to ferroptosis regulators and markers and ferroptosis-disease associations. We downloaded a total of 382 ferroptosis-related genes (FRGs) from FerrDb database, including 150 ferroptosis drivers (genes promoting ferroptosis.), 109 ferroptosis suppressors (genes preventing ferroptosis) and 123 ferroptosis markers (genes indicating the occurrence of ferroptosis). (Supplementary Table S1).

### Identification of Differentially Expressed FRGs and Functional Enrichment

The mRNA expression matrix of 382 FRGs in TCGA cohort was extracted using “limma” R package. We then utilized Wilcoxon test to filter differentially expressed FRGs (DEFRGs) between normal samples and PCa samples in TCGA cohort. The cut-off value was set as FDR (false discovery rate) < 0.05 and log2 |fold change (FC)| > 0.5. (Mock et al., 2016). All DEFRGs in PCa were shown in heatmap using “pheatmap” R package. R packages “clusterProfiler” and “org.Hs.eg.db” were utilized for perform Gene Oncology (GO) and Kyoto Encyclopedia of Genes and Genomes (KEGG) functional enrichment.

### Consensus Clustering Analysis Identifying Ferroptosis-Related Molecular Clusters

We next conducted univariable Cox regression analysis to screen prognostic DEFRGs associated with BCRFS. The cut-off *p* value was set as 0.05. Then consensus clustering analysis was performed for identifying ferroptosis-related molecular clusters using R package “ConsensusClusterPlus”.

R packages “survival”, “survminer” and “pheatmap” were used to explore the relationship of ferroptosis-related molecular subtypes with clinicopathologic characteristics (including BCRFS, T stage, N stage and age). The relationship of ferroptosis-related molecular clusters with PD-L1 gene expression level was explored. Furthermore, the ESTIMATE algorithm was utilized to evaluate tumor microenvironment (TME) scores (Ke et al., 2021), and the CIBERSORT method was used to calculate the score of 22 types of immune infiltrating cells (Chen et al., 2019). The relationships of ferroptosis-related molecular clusters with PCa immune microenvironment and immune infiltration cells were especially investigated. *p* value < 0.05 was considered statistically significant.

### Development and Verification of a Novel Ferroptosis-Based Signature for BCRFS

In this study, the TCGA cohort (405 PCa cases) was randomly split into training cohort (204 PCa cases) and testing cohort (201 PCa cases). The clinicopathological data should be comparable between the two groups. The 203 PCa cases in GEO database (GEO cohort) were used as validating cohort. We performed univariate and multivariate Cox regression analysis in training cohort to establish a novel ferroptosis-based signature for predicting BCRFS. The cutoff *p* value of univariate analysis was 0.05. On the basis of the median risk score derived from the novel ferroptosis-based signature, we divided PCa patients into high-risk and low-risk subgroup. We performed survival analysis, time-dependent the receiver operating characteristic (ROC) curve analysis and independent prognostic analysis to validate the performance of this novel ferroptosis-based signature.

More importantly, internal and external verifications were conducted in testing cohort and validating cohort, respectively. Survival analysis and time-dependent ROC curve analysis were also performed in testing cohort and validating cohort. The risk distribution of TCGA cohort, training cohort, testing cohort and validating cohort was presented using “pheatmap” R package. Especially, subgroup survival analysis was conducted in older and younger patients, stage T I-II and stage T III-IV patients, stage N0 and stage N1 patients, and different molecular subtypes. *p* value < 0.05 was considered statistically significant.

### Association of the Ferroptosis-Based Signature with Clinicopathologic Features, Tumor Stemness Scores, Immune Infiltrating Cells and Functional Enrichment.

We investigated the association of the ferroptosis-based signature with clinicopathologic features, immune cells infiltration, immune-related pathways activity and tumor stemness scores. The scores of 16 immune infiltrating cells and 13 immune function activity in TCGA cohort were calculated by single-sample gene set enrichment analysis (ssGSEA) using R package “gsva”. Moreover, gene set enrichment analysis (GSEA) was performed to investigate the underlying mechanisms related to this ferroptosis-based signature, as previous described (Cheng et al., 2021).

### Drug Sensitivity of Risk DEFRGs

CellMiner database (https://discover.nci.nih.gov/cellminer/home.do) is a database and query tool designed for the cancer research community to facilitate integration and study of molecular and pharmacological data. We applied CellMiner database to demonstrate whether these risk DEFRGs could predict the sensitivity of anticancer drugs. To enhance the relationship of ferroptosis risk genes and clinical application, only FDA approved drugs and drugs under clinical trials were included in the analysis. Spearman correlation analysis was performed to determine the correlations between the expression levels of ferroptosis risk genes and drug sensitivity.

### Validation of Risk DEFRGs Using UALCAN Database

UALCAN database (http://ualcan.path.uab.edu/) is a portal for facilitating tumor gene expression. The mRNA expression levels of risk DEFRGs between normal and tumor tissues were validated using UALCAN database.

### Cell Culture

Human PCa cell line DU145 (Procell CL-0075) and normal prostatic epithelial cell line RWPE-1(Procell CL-0200) were kindly provided by Procell Life ScienceandTechnology Co., Ltd. The DU145 cell line was cultured in MEM medium (Biological Industries, Beit HaEmek, Israel), supplemented with 10% fetal bovine serum in a standard humidified incubator at 37°C in a 5% CO2 atmosphere. The RWPE-1 cell line was cultured in RPMI-1640 medium (Biological Industries, Beit HaEmek, Israel) supplemented with 10% fetal bovine serum at 37°C in a humidified atmosphere containing 5% CO2.

### Total RNA Extraction and Quantitative Reverse-Transcription Polymerase Chain Reaction (qRT-PCR).

Most importantly, to validate the different mRNA expression levels of risk DEFRGs, we conducted qRT-PCR in DU-145 (prostate cancer cells) and RWPE-1 (normal prostatic epithelial cells) cell lines. According to the manufacturer’s protocol, the total RNA of risk DEFRGs was extracted using TRIzol reagent (Invitrogen, Carlsbad, CA). The RNA purity was detected using NanoDrop 2000 spectrometer (Thermo Fisher Scientific, Waltham, MA). Reverse transcription reactions using TransScript® Green One-Step qRT-PCR SuperMix (TransGen Biotech, Beijing, China) were conducted in only one step according to its specification. The qRT-PCR was carried out to detect the expression levels of the four genes with the Step One PlusTM PCR System (Applied Biosystems) using Taq Pro universal SYBR qPCR Master Mix (Vazyme, Nanjing, China) according to the manufacturer’s protocol. Results were normalized to the expression of GAPDH. The results of qRT-PCR were calculated by the 2^−∆∆Ct^ method: ∆Ct = Ct (risk DEFRGs)—Ct (GAPDH) and ∆∆Ct = ∆Ct (DU-145 cells)—∆Ct (RWPE-1 cells).

The primer sequences were as follows:

GAPDH: forward 5-GGT​GTG​AAC​CAT​GAG​AAG​TAT​GA-3, reverse 5-GAG​TCC​TTC​CAC​GAT​ACC​AAA​G-3, product size 123 bp;

ASNS: forward 5-CTC​CGC​GCA​GAT​CGA​ACT​AC-3, reverse 5-TCT​TTT​GGT​CGC​CAG​AGA​ATC-3, product size 197 bp;

GPT2: forward 5- CAA​GAA​GGT​GCT​GTA​CGA​GAT​G-3, reverse 5- CTC​CAT​GTA​GCC​TCC​TCT​GTA​A-3, product size 118 bp;

NFE2L2: forward 5- ATG​CCA​CAG​TCA​ACA​CAG​ATT, reverse 5-GCC​CAT​TTA​GAA​GTT​CAG​AGA​GT-3, product size 126 bp;

RRM2: forward 5-TTG​CCT​GTG​AAG​CTC​ATT​GG-3, reverse 5-CCT​CTG​ATA​CTC​GCC​TAC​TCT​C-3, product size 192 bp.

### Immunohistochemical (IHC) Staining

Three PCa tissues and BPH tissues were made into paraffin-embedded wax blocks, and then processed into 5 μM thick sections, as reported previously (Broggi et al., 2021). Briefly, after roasted at 60°C for 30 min, the tissue sections were dewaxed and hydrated. Next, 3% hydrogen peroxide was used to block the endogenous peroxidase and the citric acid buffer was used to repair antigen in boiling water for 5 min. The sections were blocked with normal sheep serum for blocking (ZLI-9022, ZSGB-BIO, Beijing, China) for 15 min and then separately incubated with the anti-ASNS (14681-1-AP, Proteintech), anti-NFE2L2 (YT3189, Immunoway), anti-RRM2 (11661-1-AP, Proteintech) and anti-GPT2 antibody (16757-1-AP, Proteintech) at 4°C overnight. After that, the secondary antibody was added for 1 h. Through DAB staining (ZLI-9017, ZSGB-BIO, Beijing, China) for 3 min, hematoxylin immersion for several minutes, differentiation liquid differentiation, and finally neutral gum sealing piece. We then used the Image J v1.51k software (https://imagej.nih.gov/ij/, Wayne Rasband, United States) to convert gray values to optical density (OD) values. After adjusting the appropriate threshold, we could get the relevant data about integrated optical density (IOD) and positive area. Finally, the average optical density (AOD) was used to assess the relative expression (AOD = IOD/area).

### Statistical Methods

Statistical analysis was performed utilizing R programming language. Univariate and multivariate Cox regression analyses were performed to establish a ferroptosis-related prognostic signature for predicting BCRFS of PCa. Univariate and multivariate independent prognostic analyses were used to demonstrate whether this ferroptosis-related prognostic signature was an independent predictor of BCRFS. Survival analysis and time-dependent the ROC curve were performed to explore the performance of this ferroptosis-related prognostic signature. *p* value < 0.05 was considered statistically significant.

## Results

### Differentially Expressed FRGs and Functional Enrichment

**FIGURE 1:**
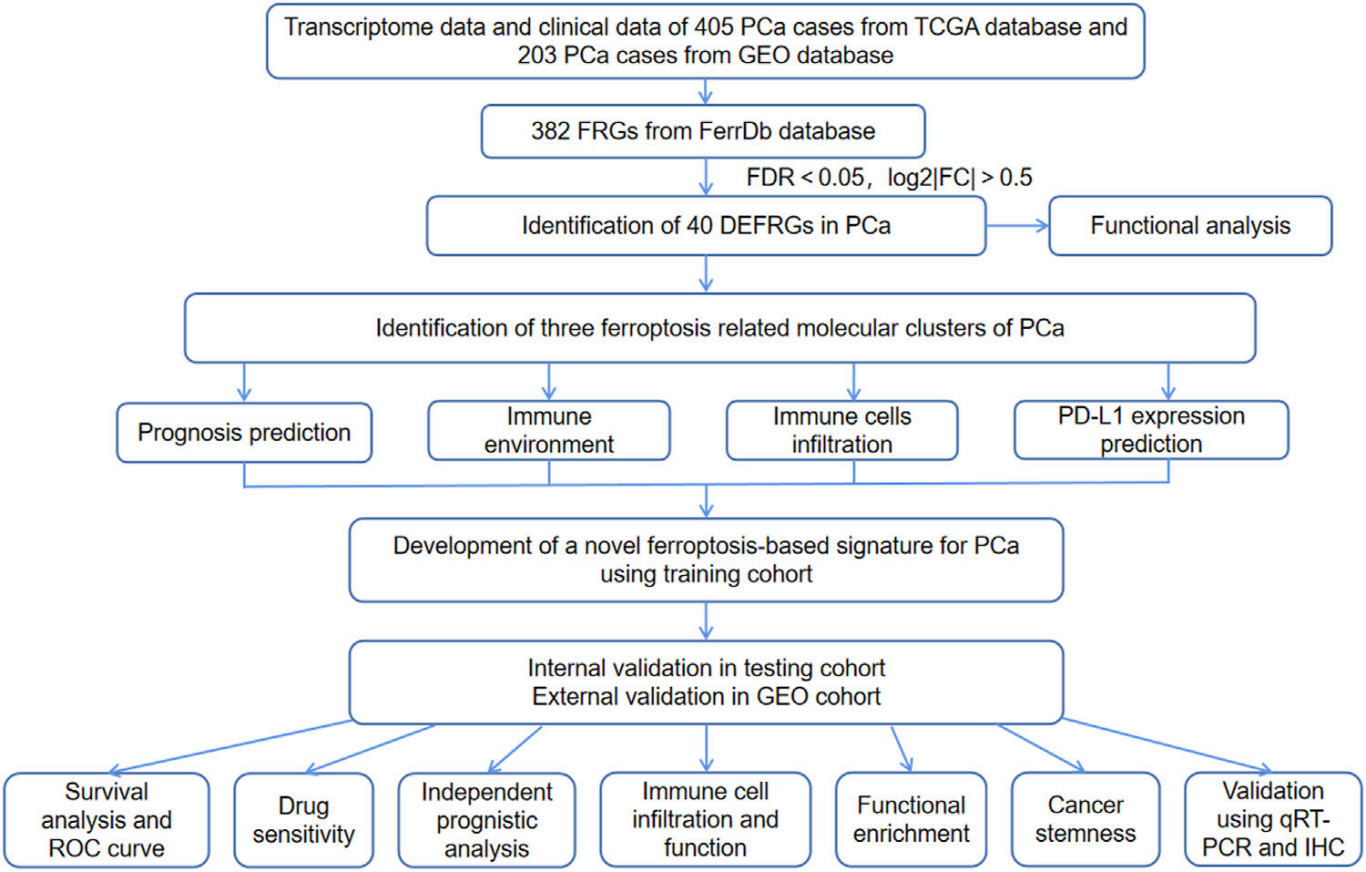
The flow chart of this study.

**FIGURE 2:**
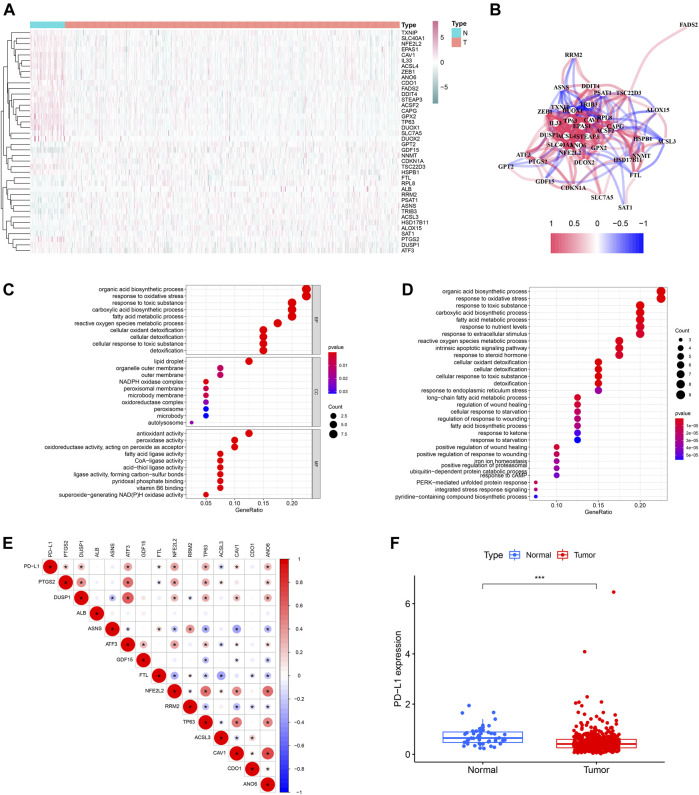
The heatmap **(A)**, correlation network **(B)** and GO **(C)** and KEGG enrichment **(D)** of 40 ferroptosis-related differentially expressed genes. The expression correlation of PD-L1 with 14 ferroptosis-related prognostic differentially expressed genes **(E)**. The expression level of PD-L1 between PCa tissue and normal tissue **(F)**.

The study flow chart was presented in Figure 1. The clinicopathologic data of training and testing cohort were showed in Table 1. Finally, a total of 40 DEFRGs were finally identified, including 26 downregulated genes and 14 upregulated genes. (Table 2). The heatmap and correlation network of these 40 FRGs were showed in Figure 2A and Figure 2B, respectively. The results of functional enrichment analysis of 40 DEFRGs were presented in Figures 2C,D. According to results of the GO functional analysis, response to oxidative stress and organic acid biosynthetic process were the main GO terms of biology process (BP); organelle outer membrane and lipid droplet were the main GO terms of cellular component (CC); peroxidase activity and antioxidant activity were the main GO terms of molecular function (MF). Moreover, KEGG analysis revealed that these 40 DEFRGs were mainly enriched in response to oxidative stress, organic acid biosynthetic process, fatty acid metabolic process, carboxylic acid biosynthetic process and response to toxic substance.

**TABLE 1:** Comparison of baseline data between training and testing cohort.

Variables	Training cohort	Testing cohort	*p* value
Age	60.87 ± 6.59	60.98 ± 6.86	0.869
T stage	—	—	0.349
T2a	1 (0.5%)	5 (2.5%)	—
T2b	4 (2.0%)	5 (2.5%)	—
T2c	62 (30.4%)	73 (36.3%)	—
T3a	69 (33.7%)	62 (30.8%)	—
T3b	62 (30.4%)	52 (25.9%)	—
T4	2 (1.0%)	3 (1.5%)	—
Unknown	4 (2.0%)	1 (0.5%)	—
N stage	—	—	0.252
N0	148 (72.6%)	142 (70.7%)	—
N1	34 (16.7%)	27 (13.4%)	—
Unknown	32 (15.7%)	32 (15.9%)	—

**TABLE 2:** Identification of differentially expressed ferroptosis related genes.

Gene	Normal mean	Tumor mean	Log2|FC|	*p* value	FDR
PTGS2	42.3182151	7.45442924	−2.50510898	1.15E-10	6.49E-10
DUSP1	261.631399	170.622883	−0.61672455	1.86E-05	4.77E-05
ALB	2.09037758	4.1752973	0.99811537	6.53E-09	2.89E-08
GPX2	13.4870552	3.92472467	−1.78091203	5.19E-20	2.70E-18
DDIT4	48.8324918	21.2101584	−1.203086	5.00E-12	3.59E-11
ASNS	5.37772335	7.77466376	0.53178474	5.08E-17	9.62E-16
TSC22D3	72.1468062	39.4798291	−0.86981979	1.16E-06	3.56E-06
TXNIP	164.004415	103.910627	−0.65839144	1.74E-08	6.96E-08
GPT2	13.8115793	20.471803	0.56775987	1.90E-06	5.49E-06
PSAT1	8.49768261	14.456719	0.7665988	2.20E-08	8.62E-08
SLC7A5	9.6259589	4.77254443	−1.01217164	7.78E-05	0.00016866
ATF3	41.0233507	22.0827061	−0.89352836	0.00017843	0.00036386
TRIB3	6.07592407	11.0007963	0.85643221	2.52E-16	4.03E-15
GDF15	21.0473355	144.794949	2.78230177	7.38E-15	7.67E-14
HSD17B11	13.1829135	20.1501115	0.61211857	0.00319137	0.00535326
FTL	766.907965	1091.81751	0.50960638	5.73E-09	2.59E-08
RPL8	395.640675	695.398594	0.8136494	5.30E-10	2.76E-09
IL33	11.8544756	6.16193743	−0.94397591	3.29E-11	2.01E-10
SLC40A1	25.1349794	13.7589342	−0.86932778	1.08E-12	8.33E-12
HSPB1	306.167341	163.477643	−0.90522705	5.78E-10	2.93E-09
NFE2L2	19.1678795	12.8732042	−0.57431956	2.86E-18	8.49E-17
STEAP3	7.41152412	5.05498478	−0.5520635	3.20E-12	2.38E-11
ALOX15	2.40403468	3.70279423	0.62315667	5.40E-06	1.50E-05
ACSF2	7.28389113	4.09436357	−0.83107014	1.95E-18	6.77E-17
NNMT	6.2166145	10.0805857	0.69737844	0.00197336	0.0035082
RRM2	2.39976615	3.47853334	0.53558532	4.52E-17	9.41E-16
CAPG	29.7087905	12.8672836	−1.20718235	5.10E-18	1.33E-16
FADS2	30.0348156	12.0841279	−1.31352245	2.63E-11	1.66E-10
TP63	11.47973	4.65738426	−1.30149689	1.59E-21	3.31E-19
CDKN1A	36.5444473	25.0585009	−0.5443521	0.00087592	0.00165629
ACSL3	24.1737903	35.5872964	0.55791864	0.00161717	0.00289975
CAV1	42.2752045	13.0398961	−1.69687936	4.77E-20	2.70E-18
DUOX1	6.81353266	3.15365595	−1.11137772	1.43E-19	5.94E-18
DUOX2	4.05444779	2.35413478	−0.78430852	6.48E-16	8.99E-15
ACSL4	7.44080417	4.40380152	−0.7567091	4.00E-16	5.95E-15
SAT1	90.4802257	157.613189	0.80071383	4.81E-07	1.63E-06
ZEB1	6.18564437	4.00813542	−0.62599264	8.33E-11	4.95E-10
CDO1	17.8257701	5.84322985	−1.60912646	2.99E-05	7.40E-05
EPAS1	28.6465826	18.5812865	−0.62451265	1.35E-08	5.60E-08
ANO6	13.6183414	6.62211382	−1.04018729	9.28E-21	9.65E-19

Univariable Cox regression analysis using the whole TCGA cohort identified a total of 14 DEFRGs related to BCRFS, including PTGS2, DUSP1, ALB, ASNS, ATF3, GDF15, FTL, NFE2L2, RRM2, TP63, ACSL3, CAV1, CDO1 and ANO6. (Table 3). The expression association of PD-L1 and these 14 FRGs was demonstrated in Figure 2E. The results showed that the expression level of PD-L1 was significantly associated with the expression level of PTGS2, DUSP1, ATF3, FTL, NFE2L2, TP63, ACSL3, CAV1, and ANO6. The expression of PD-L1 in PCa tissue was significantly decreased compared with normal tissue (Figure 2F).

**TABLE 3:** Univariable Cox regression analysis to identify DEFRGs related to biochemical recurrence-free survival in whole TCGA cohort.

Id	HR	HR.95L	HR.95H	*p* value
PTGS2	0.63419224	0.458530249	0.877149975	0.005921635
DUSP1	0.809649266	0.666766712	0.983150361	0.033047766
ALB	1.378106326	1.07978539	1.75884677	0.009974868
ASNS	3.632168303	2.137918357	6.170790635	1.84E-06
ATF3	0.819026851	0.680763127	0.985372086	0.03433078
GDF15	0.83933753	0.712762641	0.988390033	0.035730278
FTL	1.578970749	1.033805108	2.411623436	0.034530778
NFE2L2	0.608977298	0.389797411	0.951400235	0.029344571
RRM2	2.684694557	1.78713288	4.033043623	1.97E-06
TP63	0.63947479	0.441013428	0.927246157	0.01835382
ACSL3	0.702692801	0.532284759	0.927656043	0.012778296
CAV1	0.683928298	0.510882451	0.915588148	0.010695737
CDO1	0.720128798	0.532504916	0.973860465	0.033010374
ANO6	0.612113932	0.388026177	0.965613891	0.034822795

DEFRGs: differentially expressed ferroptosis related genes

### Identification of Three Ferroptosis-Related Molecular Clusters

**FIGURE 3:**
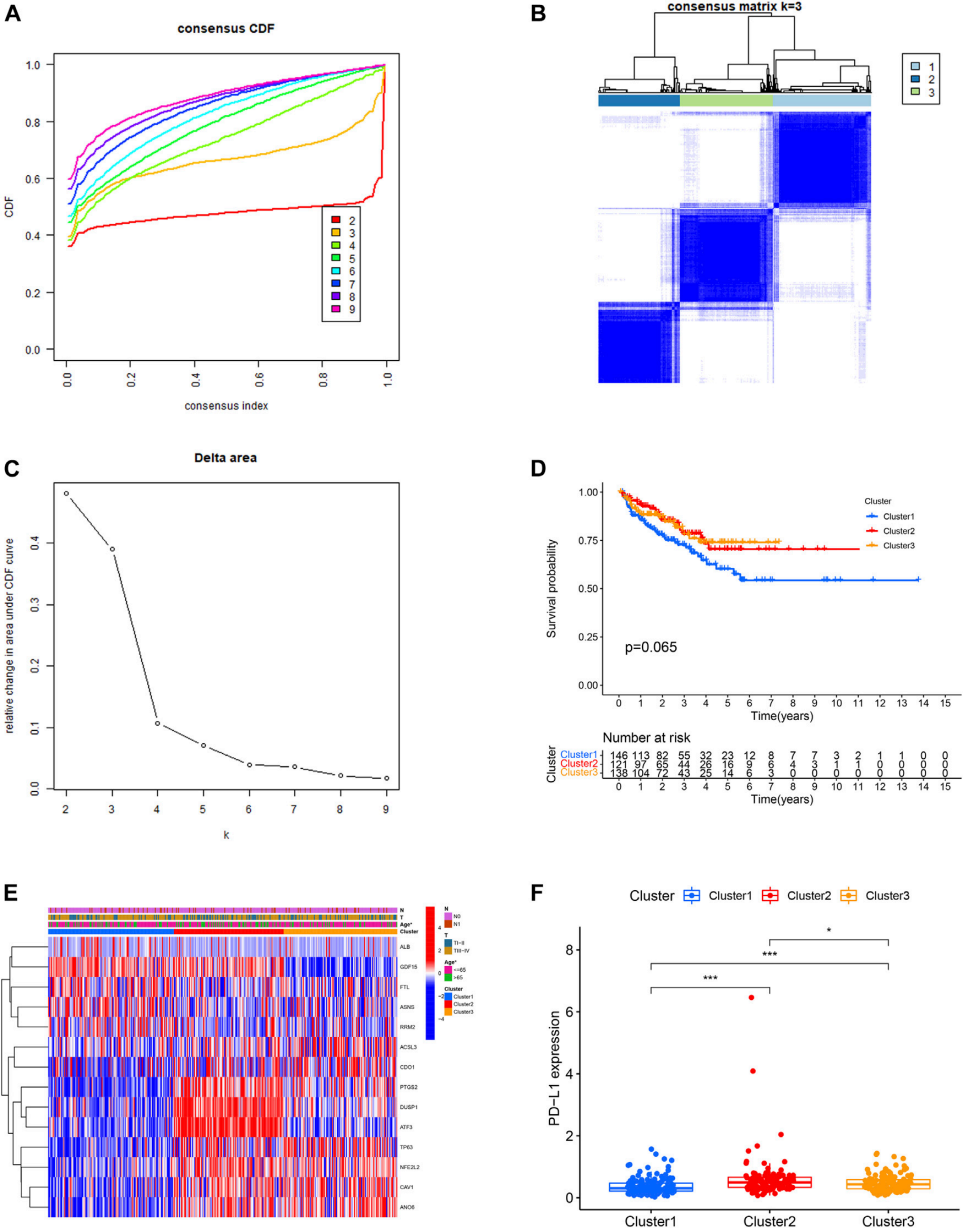
Identification of three ferroptosis-related molecular clusters using consensus clustering analysis **(A,B,C)**. Comparison of biochemical recurrence free survival among these three clusters **(D)**. The correlation heatmap between the ferroptosis-related molecular clusters and clinicopathologic features **(E)**. The expression level of PD-L1 among these three clusters **(F)**.

Then, we performed consensus clustering analysis to develop a total of three ferroptosis-related molecular clusters of PCa, including 146 cases of cluster 1, 121 cases of cluster 2, 138 cases of cluster 3 (Figures 3A–C). The difference of BCRFS among these ferroptosis-related molecular clusters was not statistically significant (Figure 3D). These three molecular clusters have significantly different age (Figure 3E).

**FIGURE 4:**
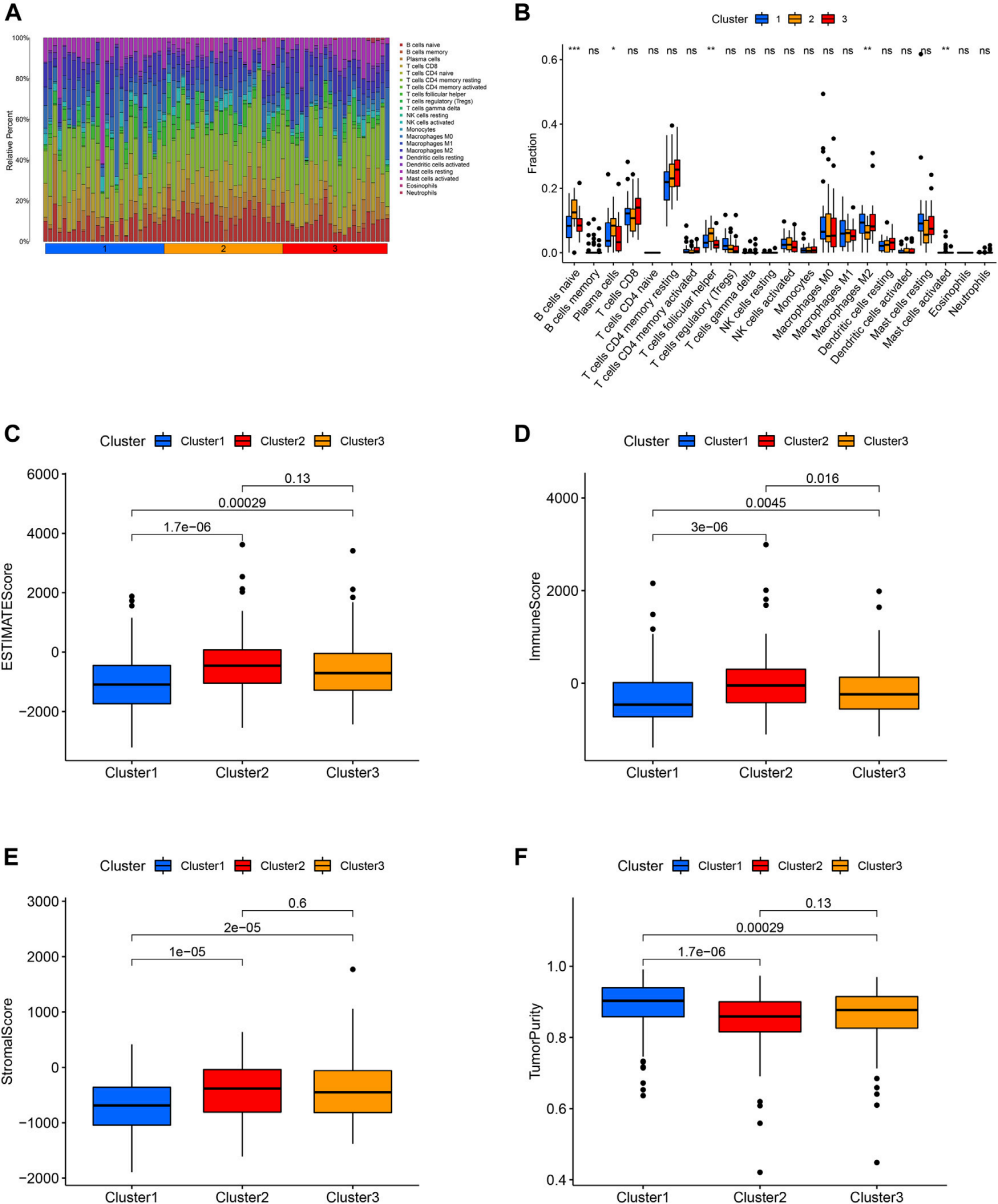
Distribution of 22 immune cell among these three molecular clusters **(A)**. Relationship between this ferroptosis-related molecular clusters and immune cell infiltration **(B)**. Relationship between the ferroptosis-related molecular clusters and ESTIMATE score **(C)**, immune score **(D)**, stromal score **(E)**, tumor purity **(F)** of tumor environment.

The expression level of PD-L1 in cluster 2 was significantly higher in comparison with that in cluster 3 and cluster 1. The expression level of PD-L1 in cluster 3 was significantly higher in comparison with that in cluster 1 (Figure 3F). The B cells naïve, plasma cells, T cell follicular helper, macrophage M2 and mast cell activated were the discrepant immune infiltrating cells among these three ferroptosis-related clusters (Figures 4A,B). The immune score in cluster 2, cluster 3 and cluster 1 was significantly decreased in sequence. The ESTIMATE score and stromal score in cluster 2 and cluster 3 were significantly increased compared with that in cluster 1; however, the ESTIMATE score and stromal score were not different between cluster 2 and cluster 3. The tumor purity in cluster 2 and cluster 3 was significantly decreased compared with that in cluster 1; however, the tumor purity was not different between cluster 2 and cluster 3. (Figures 4C–F).

### Development and Verification of a Novel Ferroptosis-Based Signature for Predicting BCRFS

**FIGURE 5:**
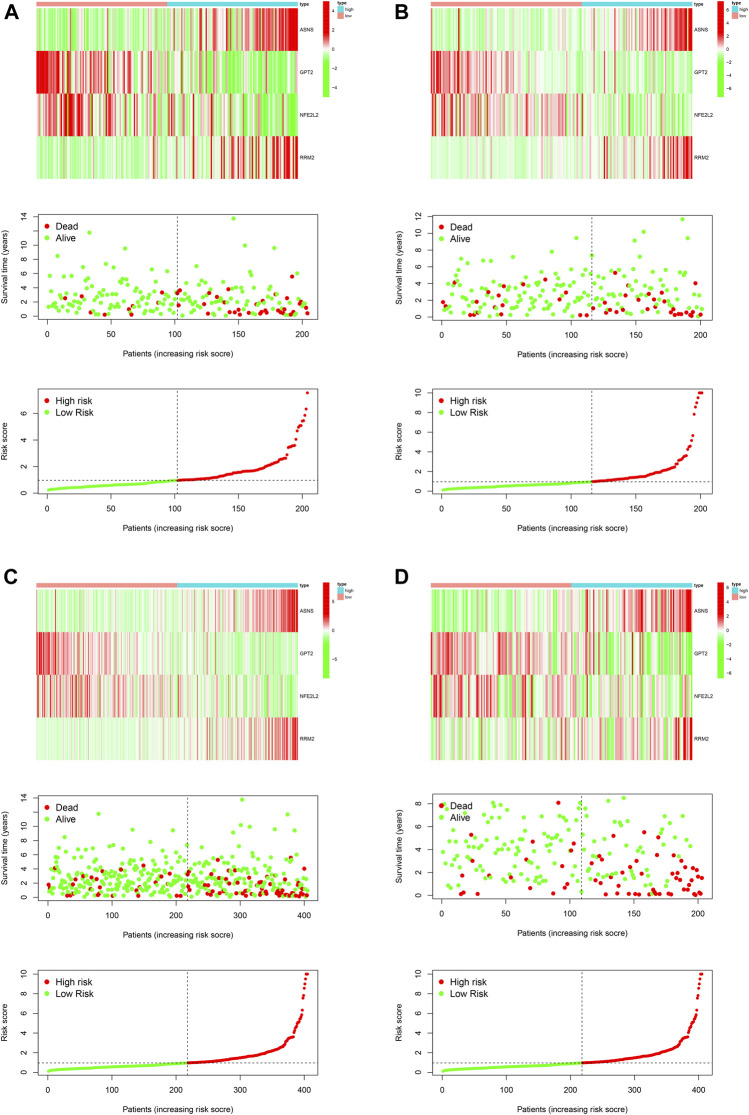
Development and validation of a novel ferroptosis-related prognostic signature for PCa. The expression heatmap, the distribution of risk score and survival time of training cohort **(A)**, testing cohort **(B)**, the whole TCGA cohort **(C)** and validating cohort **(D)**.

First of all, we performed univariable Cox regression analysis in the training cohort and screen a total of eight DEFRGs according to the threshold *p* value of 0.05 (including ASNS, GPT2, NFE2L2, RRM2, TP63, CAV1, SAT1, ZEB1). Next, based on the results of univariable analysis, we performed stepwise multivariate Cox regression analysis in training cohort to develop a novel ferroptosis-related prognostic signature and finally only four DEFRGs were included in the final model (including ASNS, GPT2, NFE2L2, RRM2). The calculating formula of risk score was as follows: risk score = (0.161502387557219) ∗ ASNS + (−0.0463523973265746) ∗ GPT2 + (−0.0709927054603633) ∗ NFE2L2 + (0.144137051044523) ∗ RRM2. (Table 4). Then, we calculated the risk score of each case using above calculation formula in training cohort, testing cohort, whole TCGA cohort and validating cohort. All patients were divided into high-risk score group and low-risk score group according to the median risk score. The distribution of survival time, risk score and the expression heatmap of training cohort, testing cohort, whole TCGA cohort and validating cohort were demonstrated in Figure 5.

**TABLE 4:** Univariate and multivariate Cox regression analysis to developing ferroptosis based prognostic signature for prostate cancer using training cohort.

Id	Univariate				Multivariate				
	HR	HR.95L	HR.95H	*p* value	coef	HR	HR.95L	HR.95H	*p* value
ASNS	1.27706302881499	1.12824773252158	1.4455069862371	0.000109368255536137	0.161502387557219	1.17527526419197	1.01991576431767	1.35430002647873	0.0255773854821206
GPT2	0.946369013315628	0.904886414541557	0.989753293862567	0.0159381399954614	−0.0463523973265746	0.954705467249913	0.911400035791957	1.00006856857852	0.0503393549213361
NFE2L2	0.892825104009218	0.819140267622551	0.973138176520924	0.00989292329156419	−0.0709927054603633	0.931468686738066	0.851922859006984	1.01844187557647	0.119058624039195
RRM2	1.24848117613746	1.08476967193504	1.43689972857474	0.00197110619212051	0.144137051044523	1.15504239744487	0.988007102847149	1.3503171546547	0.0705174582381568
TP63	0.861929089209627	0.749474176759949	0.991257307940163	0.0372446190727523	—	—	—	—	—
CAV1	0.946444888434381	0.899354930564833	0.996000462554862	0.0345262433153307	—	—	—	—	—
SAT1	1.0024041871465	1.00027866824038	1.00453422262462	0.0266069864765039	—	—	—	—	—
ZEB1	0.753157696729298	0.574133953637735	0.988003779516061	0.040646611996157	—	—	—	—	—

**FIGURE 6:**
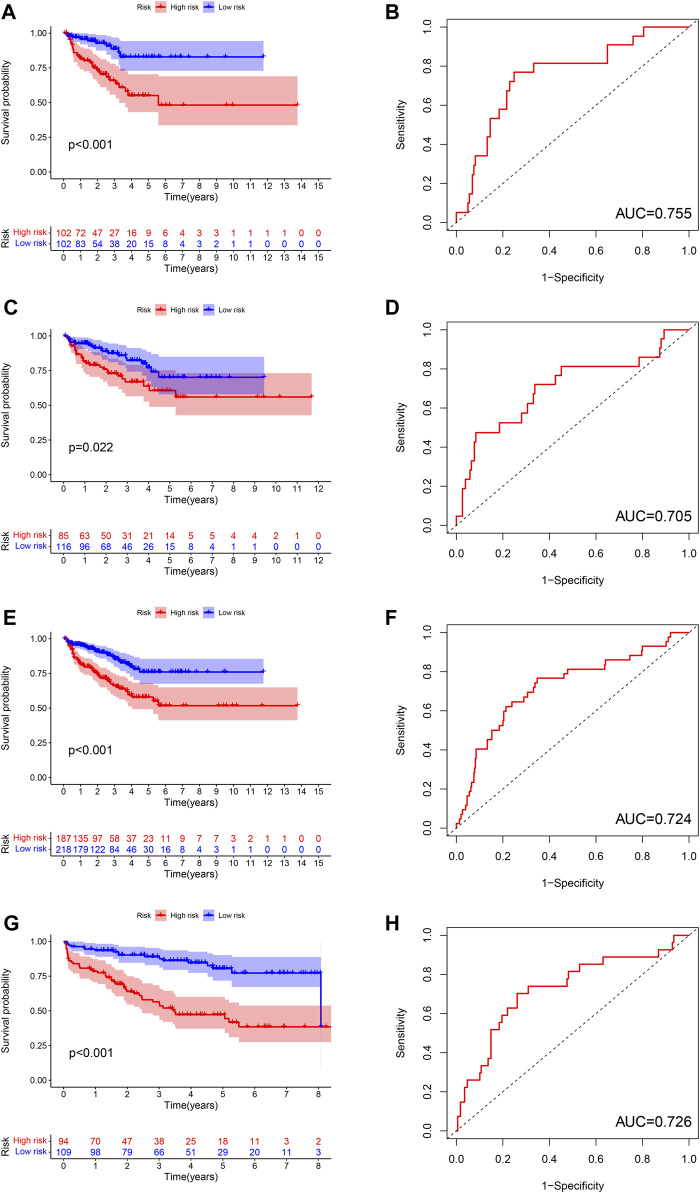
The survival analysis between high and low risk group, and corresponding area under ROC curve in training cohort **(A,B)**, testing cohort **(C,D)**, whole TCGA cohort **(E,F)**, and validating cohort **(G,H)**.

The difference of BCRFS between low-risk and high-risk subgroup was statistically significant in training cohort (*p* < 0.001), testing cohort (*p* = 0.022), whole TCGA cohort (*p* < 0.001) and validating cohort (*p* < 0.001), respectively. Patients with high-risk score were associated with significant poor BCRFS and a higher possibility of BCR risk in comparison with those with low-risk score in training cohort, testing cohort, whole TCGA cohort and validating cohort, respectively. The area under time-dependent ROC curve were 0.755, 0.705, 0.724, and 0.726 in training cohort, testing cohort, whole TCGA cohort and validating cohort, respectively, indicating the great performance of this novel ferroptosis-based signature for predicting BCRFS of PCa. (Figure 6). Univariate and multivariate independent prognostic analysis indicated that this novel ferroptosis-based signature was an independent predictor for BCRFS of PCa (Table 5, *p* < 0.05).

**TABLE 5:** Univariate and multivariate independent prognostic analysis.

Id	Univariate				Multivariate			
	HR	HR.95L	HR.95H	*p* value	HR	HR.95L	HR.95H	*p* value
Age	1.0149535013865	0.966496202058411	1.06584030830413	0.552068522390211	—	—	—	—
T	2.43085222338058	1.16943670171735	5.05289642717449	0.0173493217238285	1.75583394549311	0.797226148116186	3.86709950674698	0.162286644700018
N	2.44077937604204	1.25341796151516	4.75292691299138	0.00868414079785451	1.54365697101196	0.757596008359244	3.14531335680412	0.231881582195421
Risk score	1.47100644481883	1.22564080714238	1.76549275129281	3.39409192311108e-05	1.37429160529687	1.12688392509138	1.67601771072943	0.00169198428906916

### Subgroup Survival Analysis

**FIGURE 7:**
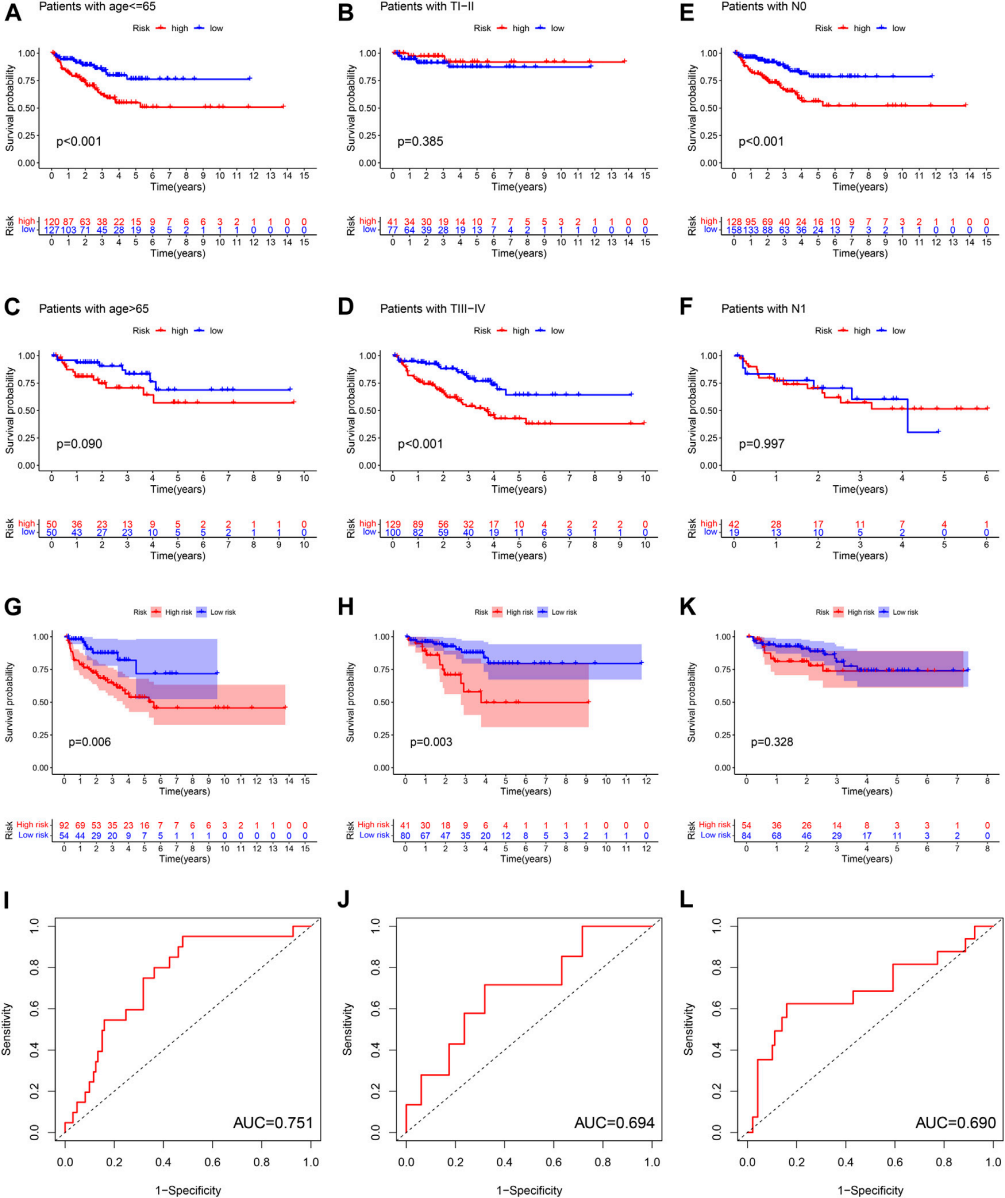
The subgroup survival analysis between high and low risk group in patients of age ≤65 **(A)**, age >65 **(B)**, T stage I-II **(C)**, T stage III-IV **(D)**, N0 stage **(E)**, N1 stage **(F)**. The survival analysis between high and low risk group, and corresponding area under ROC curve in cluster 1 cohort **(G,H)**, cluster 2 cohort **(I,J)**, cluster 3 cohort **(K,L)**.

Subgroup survival analysis revealed that this ferroptosis-based signature was particularly suitable for younger or T stage III-IV or stage N0 PCa patients. High-risk score was associated with significantly poor BCRFS compared with low-risk score in PCa cases with age≤ 65 or T stage III-IV or N0 stage. However, the difference of BCRFS between low-risk and high-risk group was not statistically significant in patients with age > 65 or T stage I-II or N1 stage (Figures 7A–F).

We calculated the risk score for each patient in three ferroptosis-related molecular clusters, respectively. The results revealed that this ferroptosis-based signature was especially applicable in cluster 1 and cluster2 PCa patients. High-risk score was associated with significantly poor BCRFS compared with low-risk score in cluster 1 and cluster 2 PCa patients. The AUC for BCRFS prediction was 0.756, 0.694 in cluster 1 and cluster 2 patients, respectively. However, the difference of BCRFS between low-risk and high-risk group was not statistically significant in cluster 3. (Figures 7G–L).

### Association of the Ferroptosis-Based Signature with Immune Cells Infiltration

**FIGURE 8:**
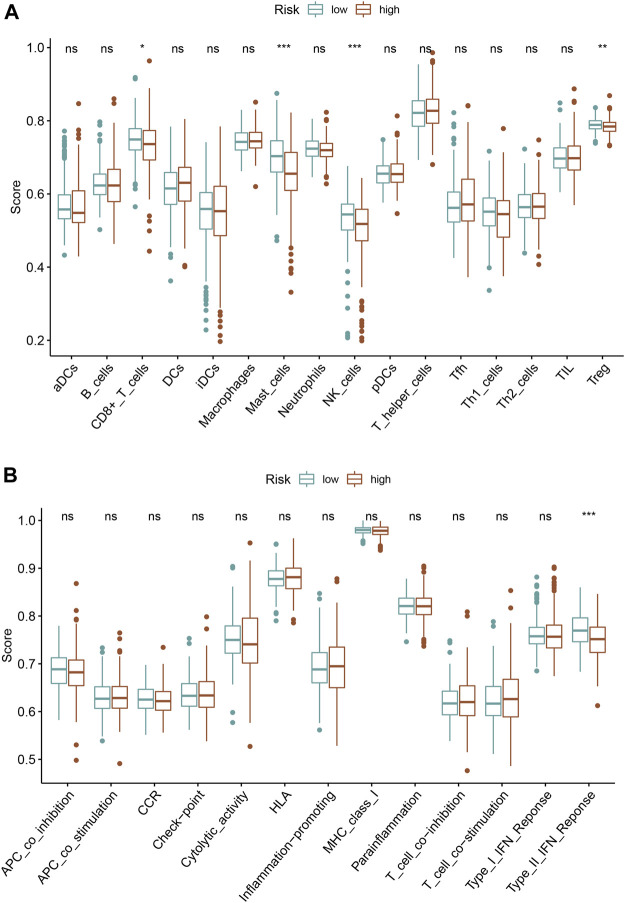
Association of the ferroptosis-based signature with immune infiltrating cells **(A)**, immune function activity **(B)**.

We quantified the immune cells infiltration and immune functions using ssGSEA to further explore the association of this ferroptosis-based signature with immune status. Interestingly, the results revealed that patients with high-risk score had a lower infiltrating proportion of CD8^+^ T cells, mast cells, NK cells, and Treg in comparison with low-risk score. Moreover, the results also demonstrated that patients with high-risk score had a lower score of type II IFN response than those with low risk (Figures 8A,B).

### Clinicopathologic Feature, Functional Enrichment and Cancer Stemness

**FIGURE 9:**
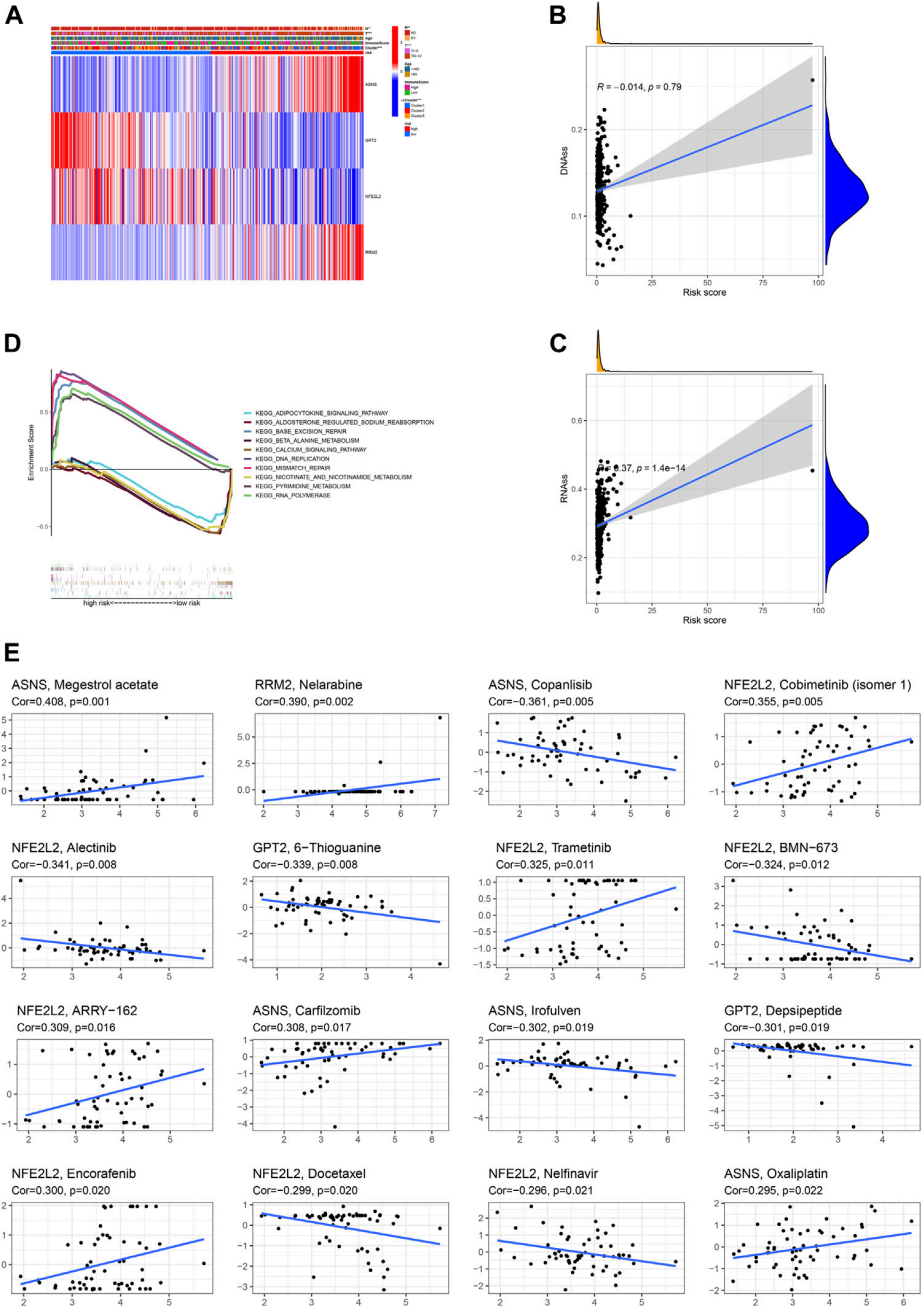
Association of the ferroptosis-based signature with clinicopathologic features **(A)**, cancer stemness **(B,C)**, functional enrichment **(D)**, anti-cancer sensitivity **(E)**.

The correlation heatmap among this prognostic ferroptosis-related signature, clinicopathologic features, ferroptosis-related molecular clusters and immune score was presented in Figure 9A. The results demonstrated that T stage, N stage, and subtypes were significantly different between high-risk patients and low-risk patients (*p* < 0.05). Besides, the correlation analysis revealed that risk score was positively associated with RNAss cancer stemness score (Figures 9B,C). KEGG functional enrichment for high-risk group and low-risk group was performed using GSEA method. RNA polymerase, base excision repair, DNA replication, pyrimidine metabolism and mismatch repair were top five KEGG enriched pathway in high-risk patients. Calcium signaling pathway, beta alanine metabolism, adipocytokine signaling pathway, nicotinate and nicotinamide metabolism and aldosterone regulated sodium reabsorption were top five KEGG enriched pathway in low-risk patients (Figure 9D).

### Drug Sensitivity of Risk DEFRGs

As revealed by Spearman correlation analysis, ASNS could predict the sensitivity of Megestrol acetate (Cor = 0.408, *p* = 0.001), Copanlisib (Cor = 0.361, *p* = 0.005), Carfilzomib (Cor = 0.308, *p* = 0.017), Irofulven (Cor = 0.302, *p* = 0.019), Oxaliplatin (Cor = 0.295, *p* = 0.022); RRM2 could predict the sensitivity of Nelarabine (Cor = 0.390, *p* = 0.002); NFE2L2 could predict the sensitivity of Cobimetinib (isomer 1) (Cor = 0.355, *p* = 0.005), Alectinib (Cor = 0.341, *p* = 0.008), Trametinib (Cor = 0.325, *p* = 0.011), BMN-673 (Cor = 0.324, *p* = 0.012), ARRY-162 (Cor = 0.309, *p* = 0.016), Encorafenib (Cor = 0.300, *p* = 0.020), Docetaxel (Cor = 0.299, *p* = 0.020), Nelfinavir (Cor = 0.296, *p* = 0.021); GPT2 could predict the sensitivity of 6-Thioguanine (Cor = 0.339, *p* = 0.008), Depsipeptide (Cor = 0.301, *p* = 0.019). (Figure 9F).

### Validation of mRNA Expression Levels of Risk DEFRGs Using UALCAN Database

**FIGURE 10:**
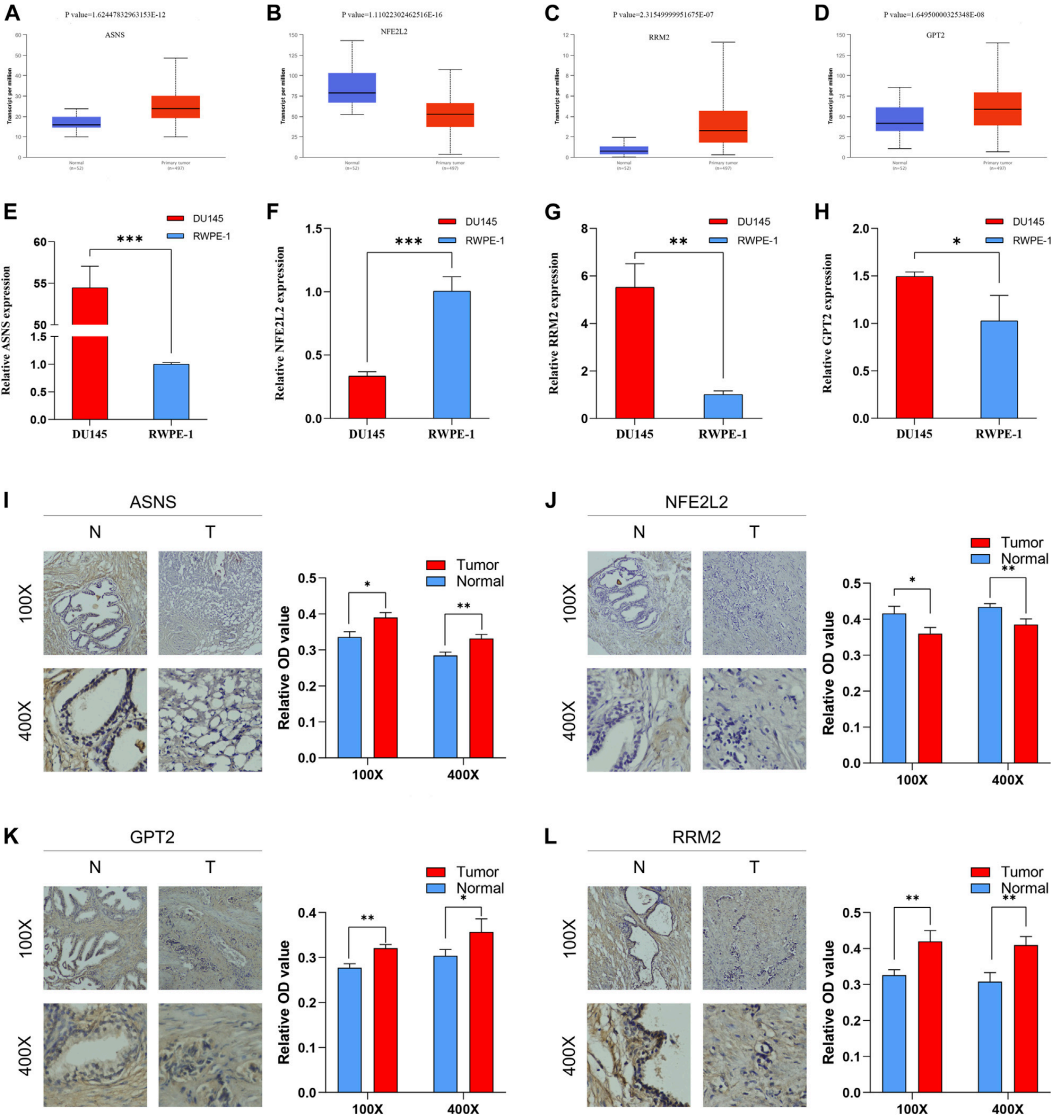
The mRNA expression levels between PCa tissue and BPH tissue of ASNS **(A)**, NFE2L2 **(B)**, RRM2 **(C)** and GPT2 **(D)**. The mRNA expression levels between PCa cells and normal prostatic epithelial cells of ASNS **(E)**, NFE2L2 **(F)**, RRM2 **(G)** and GPT2 **(H)**. The protein expression levels between BPH tissue and PCa tissue of ASNS **(I)**, NFE2L2 **(J)**, GPT2 **(K)**, RRM2 **(L)**. *p < 0.05; **p < 0.01; ***p < 0.001.

As indicated by UALCAN database, the mRNA expression levels of ASNS, GPT2, RRM2 in PCa tissue were significantly increased compared with that in normal tissue; however, the mRNA expression levels of NFE2L2 in PCa tissue was significantly decreased compared with that in normal tissue (Figures 10A–D).

### Validation of mRNA Expression Levels of Risk DEFRGs in DU-145 PCa Cells Using qRT-PCR.

The results of qRT-PCR demonstrated that the mRNA expression levels of ASNS, GPT2, RRM2 in DU-145 PCa cell lines were significantly increased compared with that in RWPE-1 normal prostatic epithelial cells; however, the mRNA expression levels of NFE2L2 in DU-145 PCa cell lines was significantly decreased compared with that in RWPE-1 normal prostatic epithelial cells (Figures 10E–H). These results were consistent with UALCAN database.

### Validation of Protein Expression Levels of Risk DEFRGs Using IHC

Moreover, the results of IHC suggested that the protein expression levels of ASNS, GPT2, RRM2 in PCa tissue were significantly increased compared with that in BPH tissue while the protein expression levels of NFE2L2 in PCa tissue was significantly decreased compared with that in BPH tissue (Figures 10I–L). These results were consistent with the results of qRT-PCR and UALCAN database.

## Discussion

Previous studies have demonstrated an important role of ferroptosis in PCa (Ghoochani et al., 2021). The induction of ferroptosis could be a novel therapeutic strategy for advanced PCa (Ghoochani et al., 2021). Therapy-induced lipid uptake and remodeling significantly promoted the hypersensitivity of ferroptosis in PCa (Tousignant et al., 2020). Nassar et al. (2020). showed that DECR1 played a vital role in protecting PCa cells from ferroptosis by regulating polyunsaturated fatty acids oxidation. BCR is considered as a key event after radical treatment of PCa (Xu et al., 2016). Despite curative treatment, there were 20–40% of PCa patients experiencing BCR with 10 years; and BCR signified recurrent PCa. (Artibani et al., 2018). Although BCR alone might have no impact on quality of life or overall survival (Wei et al., 2019), BCR indicated the high probability of the onset of metastatic disease (Malik et al., 2019). However, there were few studies exploring whether there was a correlation between ferroptosis and BCR in PCa.

In this study, we first identified a total of three molecular clusters from the perspective of ferroptosis, which were different from previous studies. These three molecular clusters exhibited diverse clinical characteristics, PD-L1 expression levels and tumor immune microenvironment features. Moreover, interestingly, we performed multivariate Cox regression analysis to develop a novel ferroptosis-based signature for predicting BCRFS of PCa based on four DEFRGs (including ASNS, GPT2, NFE2L2, RRM2). Internal and external verifications were then resoundingly performed. Patients with high-risk score were associated with significant poor BCRFS in comparison with those with low-risk score in training cohort, testing cohort, whole TCGA cohort and validating cohort, respectively. The area under time-dependent ROC curve was 0.755, 0.705, 0.724 and 0.726 in training cohort, testing cohort, whole TCGA cohort and validating cohort, respectively, indicating the great performance of this novel ferroptosis-based signature for predicting BCRFS of PCa. Univariate and multivariate independent prognostic analyses indicated that this novel ferroptosis-based signature was an independent predictor for BCRFS of PCa.

This ferroptosis-based prognostic signature was composed of four ferroptosis-related genes: asparagine synthetase (ASNS), glutamic pyruvic transaminase 2 (GPT2), nuclear factor, erythroid 2 like 2 (NFE2L2) and ribonucleotide reductase regulatory subunit M2 (RRM2). Kanishka Sircar et al. (Sircar et al., 2012). revealed that ASNS was up-regulated castration-resistant stage of prostate cancer (CRPC), and that ASNS inhibitors might be a novel method via targeting CRPC cells. Harri M Itkonen et al. (Itkonen et al., 2016). demonstrated that coinstantaneous inhibition of GPT2 and OGT would suppress the growth and viability and additionally promote death response of PCa cells. Qiang Ju et al. (Ju et al., 2020). reported that NFE2L2 might be a potential prognostic indicator and associated with immune infiltration in brain lower grade glioma. Zhang et al. (2019) indicated that RRM2 might be involved in the ferroptosis of liver cancer. Mazzu et al. (2020) showed that RRM2 could be serve as a driver of aggressive PCa and contribute to immune escape. However, whether there is a relationship between ASNS, GPT2, NFE2L2, RRM2 and PCa ferroptosis was unknown. In this study, it was found that the mRNA expression levels of ASNS, GPT2, RRM2 in PCa tissue or DU145 PCa cells were significantly increased compared with normal tissue or RWPE-1 normal prostatic epithelial cells. Besides, the mRNA expression levels of NFE2L2 in PCa tissue or DU145 PCa cells was significantly decreased compared with that in normal tissue or RWPE-1 normal prostatic epithelial cells. Moreover, the protein levels of IHC staining showed that the protein expression levels of ASNS, GPT2 and RRM2 in PCa tissue were higher than that in BPH tissue, and that the protein expression levels of NFE2L2 in PCa tissue was lower than that in BPH tissue. As revealed by Spearman correlation analysis, ASNS could predict the sensitivity of Megestrol acetate and Copanlisib; RRM2 could predict the sensitivity of Nelarabine; NFE2L2 could predict the sensitivity of Cobimetinib (isomer 1) and Alectinib; GPT2 could predict the sensitivity of 6-Thioguanine and Depsipeptide. However, the role of ferroptosis-related genes, ASNS, GPT2, NFE2L2, RRM2, in PCa ferroptosis has not been elucidated. Additional research is required.

It has been reported that tumor-induced immunosuppressive status is crucial in PCa pathogenesis (Wong and Yu, 2021). Abnormal activating inhibitory immune checkpoint pathways have been regarded as the vital method of immune escape (Modena et al., 2016). However, it is unlikely that there were long-lasting responses from immunotherapy (including various immune checkpoint inhibitor) in the whole PCa patients (Claps et al., 2020). How to identify those suitable to receive immunotherapy is hot topic of discussion for urologist (Wong and Yu, 2021). Previous studies have recognized patient subsets who might benefit from immune checkpoint monotherapies, including those with high PD-L1 expression, hypermutation or high microsatellite instability, abundant germline/somatic DNA-repair gene mutations, intraductal/ductal histology, AR-V7-positive tumors however, it was still insufficient and these indicators could not adequately reflect the state of tumor immune microenvironment (Isaacsson Velho and Antonarakis, 2018; Vitkin et al., 2019). Interestingly, in the current study, we identified a total of three ferroptosis-related molecular clusters of PCa. Further investigation revealed that the expression levels of PD-L1 in cluster 2, cluster 3 and cluster 1 were significantly decreased in sequence. The results indicated that the sensitivity to immune check point inhibitors of cluster 2, cluster 3 and cluster 1 might decrease in sequence. Furthermore, we revealed that the B cells naïve, plasma cells, T cell follicular helper, macrophage M2 and mast cell activated were the discrepant immune infiltrating cells among these three ferroptosis-related clusters. The immune scores in cluster 2, cluster 3 and cluster 1 were also significantly decreased in sequence. The ESTIMATE score and stromal score in cluster 2 and cluster 3 were significantly increased compared with that in cluster 1. The tumor purity in cluster 2 and cluster 3 was significantly decreased compared with that in cluster 1. Patients with high-risk score have a lower infiltrating proportion of CD8^+^ T cells, mast cells, NK cells, and Treg and a lower score of type II IFN response in comparison with those with low-risk score.

Several limitations should be noted in this study. First of all, retrospective design with limited sample size would result in selection bias. Prospective real-world data are required to verify this ferroptosis-based molecular signature due to the retrospective methods of this studies. Besides, although qRT-PCR and IHC has been performed to validate the different mRNA and protein expression levels of risk DEFRGs, further wet experiment *in vitro* and *in vivo* into the underlying mechanism related to the correlation of ferroptosis with PCa immunotherapy and BCR are warranted.

## Conclusion

This study identified a total of 40 differentially expressed FRGs and three ferroptosis-related molecular clusters in PCa. More importantly, a novel ferroptosis-based prognostic molecular signature of PCa based on four FRGs (including ASNS, GPT2, NFE2L2, RRM2) was developed, which has the great performance for predicting BCRFS. Our findings would contribute tremendously to the potential mechanism in PCa occurrence and development.

## Data Availability

The original contributions presented in the study are included in the article/Supplementary Material, further inquiries can be directed to the corresponding authors.
