# Supplementary material for: A Novel Ferroptosis-Based Molecular Signature Associated with Biochemical Recurrence-Free Survival and Tumor Immune Microenvironment of Prostate Cancer
Source: Front Cell Dev Biol. 2022 Jan 6;9:774625. doi: 10.3389/fcell.2021.774625 (PMC8773967; doi:10.3389/fcell.2021.774625)
Supplement: Supplementary file 1 [file Table1.docx]

**Supplemental Table 1. The list of ferroptosis related genes**

PTGS2

DUSP1

NOS2

NCF2

MT3

UBC

ALB

TXNRD1

SRXN1

GPX2

BNIP3

OXSR1

SELENOS

ANGPTL7

CHAC1

SLC7A11

DDIT4

LOC284561

ASNS

TSC22D3

DDIT3

JDP2

SESN2

SLC1A4

PCK2

TXNIP

VLDLR

GPT2

PSAT1

LURAP1L

SLC7A5

HERPUD1

XBP1

ATF3

SLC3A2

CBS

ATF4

ZNF419

KLHL24

TRIB3

ZFP69B

ATP6V1G2

VEGFA

GDF15

TUBE1

ARRDC3

CEBPG

SNORA16A

RGS4

BLOC1S5-TXNDC5

LOC390705

EIF2S1

KIM-1

IL6

CXCL2

RELA

HSD17B11

AGPAT3

SETD1B

HMOX1

TF

FTL

RPL8

ATP5MC3

TFRC

MAFG

IL33

FTH1

SLC40A1

TF

TFRC

FTH1

GPX4

HAMP

HSPB1

NFE2L2

STEAP3

DRD5

GPX4

DRD4

MAP3K5

MAPK14

SLC2A1

SLC2A3

SLC2A6

SLC2A8

SLC2A12

GLUT13

SLC2A14

EIF2AK4

EIF2S1

ATF4

ALOX5

ALOX12

ALOX15

ALOX5

ACSF2

IREB2

GPX4

HMGB1

HMOX1

NFE2L2

ELAVL1

SLC3A2

SLC7A11

TFAP2C

SP1

HBA1

NNMT

PLIN4

HIC1

STMN1

RRM2

CAPG

HNF4A

NGB

YWHAE

GABPB1

AURKA

MIR4715

RIPK1

PRDX1

MIR30B

SLC7A11

GPX4

AKR1C1

AKR1C2

AKR1C3

GPX4

RB1

HSPB1

HSF1

SLC7A11

GPX4

GCLC

SLC7A11

NFE2L2

SQSTM1

NQO1

HMOX1

FTH1

MUC1

SLC3A2

MT1G

NFE2L2

SLC40A1

SLC7A11

GPX4

SLC7A11

CISD1

SLC7A11

FANCD2

GPX4

NFE2L2

FTMT

HSPA5

ATF4

SLC7A11

GPX4

GPX4

HMOX1

ATF4

NFE2L2

TP53

SLC7A11

HELLS

SCD

FADS2

SRC

STAT3

NFE2L2

PML

MTOR

NFS1

TP63

SLC7A11

TP53

CDKN1A

MIR137

SLC40A1

GPX4

GPX4

ENPP2

VDAC2

FH

CISD2

SLC40A1

MIR9-1

MIR9-2

MIR9-3

CBS

NFE2L2

SQSTM1

GPX4

ISCU

FTH1

ACSL3

OTUB1

CD44

LINC00336

STAT3

BRD4

PRDX6

MIR17

SCD

SESN2

NF2

ARNTL

HIF1A

JUN

CA9

HSPA5

TMBIM4

HSPA5

PLIN2

MIR212

Fer1HCH

AIFM2

AIFM2

LAMP2

ZFP36

GPX4

PROM2

CHMP5

CHMP6

AKR1C1

AKR1C2

AKR1C3

CBS

NFE2L2

CAV1

GCH1

RPL8

IREB2

ATP5MC3

CS

EMC2

ACSF2

NOX1

CYBB

NOX3

NOX4

NOX5

DUOX1

DUOX2

G6PD

PGD

VDAC2

PIK3CA

FLT3

SCP2

TP53

ACSL4

LPCAT3

NRAS

KRAS

HRAS

TF

TFRC

TFR2

SLC38A1

SLC1A5

GLS2

GOT1

CARS1

TP53

ALOX5

KEAP1

HMOX1

TP53

TP53

GLS2

ATG5

ATG7

NCOA4

TF

ALOX5

ALOX12

ALOX12B

ALOX15

ALOX15B

ALOXE3

PHKG2

TFRC

ACO1

IREB2

SLC38A1

GLS2

G6PDX

ULK1

ATG3

ATG4D

ATG5

BECN1

MAP1LC3A

GABARAPL2

GABARAPL1

ATG16L1

WIPI1

WIPI2

SNX4

ATG13

ULK2

NCOA4

ACSL4

TP53

SAT1

ALOX15

ACSL4

LPCAT3

ALOX15

ACSL4

KEAP1

EGFR

NOX4

MAPK3

MAPK1

BID

ACSL4

ZEB1

KEAP1

DPP4

ALOX15

ALOX12

CDKN2A

PEBP1

SOCS1

CDO1

MYB

HMOX1

MAPK8

MAPK9

MAPK1

MAPK3

SLC1A5

CHAC1

MAPK14

LINC00472

NOX4

GOT1

BECN1

PRKAA2

PRKAA1

ELAVL1

BAP1

TP53

ABCC1

ACSL4

MIR6852

ACVR1B

TGFBR1

BAP1

EPAS1

HILPDA

HIF1A

ALOX12

ACSL4

HMOX1

IFNG

ANO6

LPIN1

HMGB1

TNFAIP3

TLR4

NOX4

ATF3

ATM

YY1AP1

EGLN2

MIOX

TAZ

MTDH

IDH1

SIRT1

TAZ

BECN1

FBXW7

PANX1

DNAJB6

BACH1

ACSL4

LONP1
